# Reviewer Acknowledgement 2013

**DOI:** 10.1186/2046-4053-3-6

**Published:** 2014-01-28

**Authors:** Daniel Shanahan

**Affiliations:** 1 BioMed Central, 236 Gray's Inn Road, London, WC1X 8HB, United Kingdom

## Abstract

**Contributing reviewers:**

A peer-reviewed journal would not survive without the generous time and insightful comments of the reviewers, whose efforts often go unrecognized. *Systematic Reviews* has been blessed by the support of highly-qualified peer reviewers, and the Editors-in-Chief, David Moher, Lesley Stewart and Paul Shekelle, and staff of the journal would like to show their appreciation by thanking the following for their invaluable assistance with review of manuscripts for the journal in Volume 2 (2013).

## 

Ahmed Abou-Setta

Egypt

Tony Ades

United Kingdom

Jesmin Antony

Canada

Luis F Azevedo

Portugal

Ethan Balk

United States of America

Joel Bamford

United States of America

Emmanuelle Belanger

Canada

Julia Betts

United Kingdom

Laura Bonnett

United Kingdom

Andrew Booth

United Kingdom

Kenneth Brown

United States of America

Brad Carlin

United States of America

Kathleen Carlson

United States of America

Chris Carswell

New Zealand

Anna Chaimani

Greece

Stephanie Chang

United States of America

Alexander Clark

Canada

Ryan Coller

United States of America

Gary Cooney

United Kingdom

Esther Coren

United Kingdom

Miranda Cumpston

Australia

Isabel De Salis

United Kingdom

Declan Devane

Ireland

Sanket Dhruva

United States of America

Sofia Dias

United Kingdom

Marjolein Dieleman

Netherlands

Janine Dretzke

United Kingdom

Jo Dumville

United Kingdom

Kerry Dwan

United Kingdom

Tarig Elraiyah

United States of America

Jonathan Emberson

United Kingdom

Zbys Fedorowicz

Bahrain

Finola Ferry

United Kingdom

Elizabeth Fitzpatrick

Canada

Alan Fleischer

United States of America

Nathan Ford

Switzerland

Ruth Foxlee

United Kingdom

Constance Fung

United States of America

Rohan Ganguli

Canada

Chantelle Garritty

Canada

Laura Gaudet

Canada

Jean-Francois Gehanno

France

Michael Goldstein

United States of America

Joanne Greenhalgh

United Kingdom

Ulrich Grouven

Germany

Shirin Heidari

Switzerland

Morag Heirs

United Kingdom

Susanne Hempel

United States of America

David Howells

Australia

Carys Jones

United Kingdom

Kathryn Kaiser

United States of America

Devan Kansagara

United States of America

Jamie Kirkham

United Kingdom

Werner Kulp

Germany

Tom Lang

United States of America

Dean Langan

United Kingdom

Toby Lasserson

United Kingdom

Joseph Lau

United States of America

Mariska M.G. Leeflang

Netherlands

Alexis Llewellyn

United Kingdom

Margaret Maglione

United States of America

Sabine Maguire

United Kingdom

Norman Marks

United States of America

Rachel Marshall

United Kingdom

Homero Martinez

United States of America

Jessie McGowan

Canada

Nick Meader

United Kingdom

Joerg J. Meerpohl

Germany

Edward Mills

Canada

Jens Minnerup

Germany

David Moher

Canada

David Moore

United Kingdom

Reg Morris

United Kingdom

Andra Morrison

Canada

Cynthia Mulrow

United States of America

Sarah Nolan

United Kingdom

Charles Normand

Ireland

Susan L. Norris

United States of America

Maya O'Neil

United States of America

Kaat Peerenboom

Belgium

Frank Peinemann

Germany

Nicola Petrucci

Italy

Mark Petticrew

United Kingdom

Bob Phillips

United Kingdom

Wilfred Pigeon

United States of America

Shalea Piteau

Canada

Robert Platt

Canada

Marcel Post

Netherlands

Malcolm Price

United Kingdom

David Riley

United States of America

Mark Rodgers

United Kingdom

Gerta Rücker

Germany

Audrey Saftlas

United States of America

Margaret Sampson

Canada

Holger Schünemann

United States of America

Rob Scholten

Netherlands

Georg M. Schuetz

Germany

Paul Shekelle

United States of America

Jonathan Shepherd

United Kingdom

Mark Simmonds

United Kingdom

Becky Skidmore

Canada

Nuno Sousa

Portugal

Mai Stafford

United Kingdom

Johann Steurer

Switzerland

Adrienne Stevens

Canada

Gavin Stewart

United Kingdom

Xin Sun

Canada

Karl Sweeney

Ireland

Yemisi Takwoingi

United Kingdom

Britta Tendal

Denmark

David Tovey

United Kingdom

Lyndal Trevena

Australia

Guy Tsafnat

Australia

Alexander Tsertsvadze

Canada

Wynanda Van Enst

Netherlands

Arianne Verhagen

Netherlands

Christiane Voisin

United States of America

Vivian Welch

Canada

Emma Welsh

United Kingdom

Shi Wu Wen

Canada

Marie Elaine Westwood

United Kingdom

Donna White

United States of America

Penny Whiting

United Kingdom

Simon Wieser

Switzerland

Kath Wright

United Kingdom

Wei-Feng Yu

China

Babalwa Zani

South Africa

Paula Ziegler

United States of America

